# The Usefulness of Carotid Artery Doppler Measurement as a Predictor of Early Death in Sepsis Patients Admitted to the Emergency Department

**DOI:** 10.3390/jcm13226912

**Published:** 2024-11-16

**Authors:** Su-Il Kim, Yun-Deok Jang, Jae-Gu Ji, Yong-Seok Kim, In-Hye Kang, Seong-Ju Kim, Seong-Min Han, Min-Seok Choi

**Affiliations:** 1 Department of Paramedicine, Sunlin University, Pohang 37560, Republic of Korea; avantetop@hanmail.net; 2 Department of Emergency Medicine, Inje University Busan Paik Hospital, Busan 47392, Republic of Korea; ws1234@naver.com; 3 Department of Paramedicine, Konyang University, Daejeon 32992, Republic of Korea; ys031113@konyang.ac.kr; 4 Department of Paramedicine, Daewon University College, Jecheon 27135, Republic of Korea; emtkih@hanmail.net; 5 Department of Paramedicine, Tongmyong University, Busan 48520, Republic of Korea; superemt@naver.com; 6 Department of Paramedicine, Seoyeong University, Gwangju 61268, Republic of Korea; han@seoyeong.ac.kr; 7 Department of Paramedicine, Yeungnam University, Gyeongsan 38541, Republic of Korea; dreebok@naver.com

**Keywords:** sepsis, carotid artery, Doppler measurement, ultrasonography, emergency department

## Abstract

**Background:** This study aims to verify whether the blood flow velocity and the diameter size, measured through intra-carotid artery Doppler measurements performed on sepsis patients visiting the emergency department, are useful as tools for predicting the risk of early death. **Methods:** As a prospective study, this research was performed on sepsis patients who visited a local emergency medical center from August 2021 to February 2023. The sepsis patients’ carotid artery was measured using Doppler imaging, and they were divided into patients measured for the size of systolic and diastolic mean blood flow velocity and diameter size: those measured for their qSOFA (quick sequential organ failure assessment) score and those measured using the SIRS (systemic inflammatory response syndrome) criteria. By measuring and comparing their mortality prediction accuracies, this study sought to verify the usefulness of blood flow velocity and the diameter size of the intra-carotid artery as tools to predict early death. **Results:** This study was conducted on 1026 patients, excluding 45 patients out of the total of 1071 patients. All sepsis patients were measured using systolic and diastolic blood flow velocity and diameter by Doppler imaging of the intra-carotid artery, assessed using qSOFA and evaluated using SIRS criteria. The results of the analysis performed to compare the mortality prediction accuracy were as follows. First, the hazard ratio (95% CI) of the intra-carotid artery was significant (*p* < 0.05), at 1.020 (1.004–1.036); the hazard ratio (95% CI) of qSOFA was significant (*p* < 0.05), at 3.871 (2.526–5.931); and the hazard ratio (95% CI) of SIRS showed no significant difference, at 1.002 (0.995–1.009). After 2 h of infusion treatment, the diameter size was 4.72 ± 1.23, showing a significant difference (*p* < 0.05). After 2 h of fluid treatment, the blood flow velocity was 101 m/s ± 21.12, which showed a significant difference (*p* < 0.05). **Conclusions:** Measuring the mean blood flow velocity in the intra-carotid arteries of sepsis patients who visit the emergency department is useful for predicting the risk of death at an early stage. And this study showed that Doppler measurement of the diameter size of the carotid artery significantly increased after performing fluid treatment after early recognition.

## 1. Introduction

In sepsis patients, overall circulation decreases due to a decrease in stroke volume (SV) [1]. As the overall circulation decreases, hemodynamic changes appear and the patient dies [2]. Therefore, early risk recognition is important, and hemodynamic monitoring is important while treating patients [3]. However, in fact, there are many difficulties in confirming circulation through SV measurement in sepsis patients. Recently, the advantages of carotid Doppler measurement, as a non-invasive hemodynamic monitoring tool, on several severe emergency patients, have been emphasized [4]. Carotid artery Doppler measurement is a useful device that can non-invasively observe clinical changes in patients by measuring the blood flow velocity and diameter size of the carotid artery [5]. In particular, in critically ill emergency patients, stroke volume (SV) is a useful hemodynamic monitoring tool [4]. Hypovolemia and cardiac filling with hemodynamic changes result in decreased SV, and compensatory adrenergic responses result in changes that increase systemic vascular resistance [4,5]. In addition, carotid Doppler ultrasound measurements have been performed to check cerebral blood flow in shock patients and cardiac arrest patients, and these non-invasive hemodynamic tools have been proven effective as hemodynamic monitoring tools for severe emergency patients [5].

Sepsis refers to a systemic syndrome of physiological, biological, and biochemical abnormalities due to a dysregulated host response to an infection. Currently, there is no straightforward treatment for sepsis and, eventually, multiple organ failure syndromes (MOFSs) result in death. Sepsis has high rates of mortality and causes chronic illness worldwide, and the disease is commonly seen in intensive care units (ICUs). Over the past several decades, the understanding of its causes and pathophysiological foundations continued to evolve, providing evidence to support the use of sepsis treatment guidelines [6].

According to what has been discovered so far, the occurrence of sepsis is higher in people of age 65 or older and in individuals with immunosuppression, multidrug-resistant bacterial infection, diabetes, obesity, cancer, and bacteremia [7,8,9]. Although it varies depending on race and ethnicity, the occurrence is the highest in male Americans of African descent [10]. Sepsis occurs most commonly in the winter, which is likely due to an increase in respiratory infection [11].

Presently, sepsis is explained in terms of the production of anti-inflammatory cytokines due to the release of pro-inflammatory cytokines. The pathogens of sepsis identified so far include bacteria, fungi, and viruses. According to Mayer et al. [12,13,14], as deeper insight was gained into post-infection pathological mechanisms, recent studies reported a few new host response models.

Bacteria are known as a primary source of infection in sepsis. Of the bacteria causing infection, Gram-positive bacteria are reported to be most common in sepsis patients in the US. However, a considerable number of cases of sepsis are due to Gram-negative bacteria. The occurrence of fungal sepsis has increased in the past decade, although the level is lower in comparison to bacterial sepsis [10,15]. COVID-19, which caused the current pandemic, is a representative example of a respiratory virus that causes severe illness. Additional examples are severe acute respiratory syndrome coronavirus (SARS-CoV) and Middle East Respiratory Syndrome (MERS) [16].

The primary clinical signs of sepsis are a body temperature of >38.3 or <36 °C, changed GCS, hypotension (systolic blood pressure of <90 mmHg, mean arterial pressure (MAP) of <70 mmHg, a reduction in systolic blood pressure by >40 mmHg, or systolic blood pressure of less than 2 SD of the normal value for age), tachypnea (over 20/min), and a heart rate of over 90/min. Changes in blood test results specific to sepsis include the following: elevated or decreased levels of white blood cells; elevated blood sugar levels in the absence of diabetes (plasma glucose level >140 mg/dL or 7.7 mmol/L); C-reactive protein (CRP) levels that are higher than normal; arterial hypoxia; acute oliguria; increased creatinine (>0.5 mg/dL); thrombocytopenia; hyperbilirubinemia (total plasma bilirubin > 4 mg/dL); lactic acidosis; and acute oliguria [17].

Even today, treatment guidelines continue to be developed for sepsis patients with these life-threatening signs. Despite the vast volume of research over many decades, however, there was no treatment specific to sepsis. Only early diagnosis is known to be critical in treating sepsis [18]. An early diagnosis can make it possible to eliminate the source of infection early, administer antibiotics promptly, and stabilize hemodynamic status, thus greatly improving the survival rate of sepsis patients [19].

Accordingly, the Surviving Sepsis Campaign guidelines (SSC) are used worldwide to increase early recognition of sepsis and decrease the mortality rate. The SSC guideline was created in 2002 to contribute to the survival of sepsis patients through early recognition and prompt treatment [20]. Currently, various clinical scoring systems based on clinical signs are used to recognize sepsis early. Quick Sequential Organ Failure Assessment (qSOFA) and the systemic inflammatory response syndrome criteria (SIRS criteria) are commonly used tools [21,22].

Sepsis is commonly seen in the ED, too. The prevalence is 266–453 per 100,000 population, and the mortality reaches 27–30% [23]. In recent studies, the existing tools for early risk assessment were applied to patients admitted to the ED [24]. Nevertheless, there is little evidence for using predictive tools for early risk assessment in the ED, and it is necessary to develop a practical and accurate tool to predict sepsis early in ED patients [25,26]. The reason is that qSOFA or SIRS is measured directly by medical staff, so subjective thoughts are involved, and there are limitations in using it as an objective indicator [27,28,29]. In addition, if sepsis patients who visit the emergency room visit in the early stage, there is a problem that qSOFA or SIRS points may be measured as a somewhat normal patient condition. Therefore, it is necessary to confirm the progress of sepsis in emergency room patients with objective clinical indicators [30].

The objectives of the study were as follows. The first objective was to demonstrate the usefulness of carotid Doppler ultrasound as a predictive tool to assess the early risk of death by measuring blood flow velocity in the intra-carotid artery (ICA) and the artery diameter in sepsis patients with hypotension among those admitted in the ED. Studies conducted so far tested the usefulness of the tools developed for ICU and general ward patients in assessing early risk in sepsis patients in the ER. Of those, qSOFA and SIRS were used in this study to compare with ICA blood flow velocity and diameter measured with Doppler ultrasound, on the accuracy of predicting death.

In this study, however, the authors aimed to investigate whether ICA blood flow velocity and diameter, estimated by applying the new predictive tool for death, Doppler ultrasound, could be used as predictive factors for recognizing early death.

## 2. Materials and Methods

### 2.1. Data Collection

This prospective study was conducted based on the medical records and Doppler ultrasound of patients diagnosed with or suspected sepsis from November 2021 to November 2023. Patients or their legal guardians were interviewed to provide consent after being fully informed of the study procedures, objectives, and potential risks, including any complications associated with ultrasound. Written consent was obtained accordingly. The present study was approved by the institutional review board (IRB) of Inje University Busan Paik Hospital (Busan, Republic of Korea; IRB No. 2021-11-063).

They were fully informed by an ED physician of the study procedure and objectives. Additionally, they were advised that ultrasound was harmless to the body and that certain complications could occur.

Data were collected in two ways. First, the following data were obtained from the medical record such as general characteristics, including age and the presence or absence of underlying diseases, the location of the infection (respiratory system, urinary system, digestive system, skin, soft tissues, and all other locations), death (within 30 days), survival, the Korean Triage and Acuity Scale (KTAS), heart rate, blood pressure (systolic and diastolic blood pressure) upon arrival at the ED, body temperature, end-tidal CO_2_ (ETCO_2_), Glasgow coma scale (GCS) (<15), the use of antibiotics before the arrival at the ED (within 12 h after the onset of illness) in transferred patients, the number of days on mechanical ventilation, the status of application of the Surviving Sepsis Campaign guidelines (fluid therapy, antibiotics, blood culture test, and 2 or more lactic acid tests), the number of days in the ICU, and total duration of hospital stay. The following blood test results were obtained by reviewing the medical charts, including arterial blood gas analysis (ABGA) for PaO2, PACO2, and the base excess extracellular fluid, as well as hematological indices for diagnosing sepsis (C protein, procalcitonin, and lactic acid).

In addition, classifications based on the scores of the tools commonly used for early risk assessment in sepsis patients, namely, Acute Physiology and Chronic Health Evaluation II (APACHE), qSOFA, and SIRS criteria, were performed using MedCalc ver. 12.7.0 (MedCalc. Software, Ostend, Belgium).

To evaluate whether the presence or absence of delirium, time of death, censored data value, the history of ICU admission, duration of hospital stays, and survival status at discharge would be useful in predicting death, the relevant data were collected.

### 2.2. Procedures

**Figure 1:**
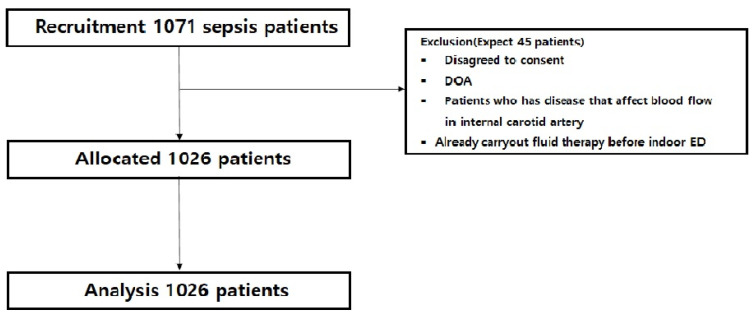
Algorithm of study flow. DOA: death on arrival; ED: emergency department; qSOFA: quick sequential organ failure; SIRS criteria: systemic inflammatory response syndrome criteria.

Of the total 1071 patients recruited for the study, 45 were excluded, and the remaining 1026 were recruited [31]. Among 45 patients who did not write a consent form, those who were dead at the time of visit, had underlying diseases that could cause carotid artery blood flow problems, or visited from other hospitals to receive fluid therapy were excluded, and this study was conducted on 1026 patients [29,32]. Specifically, patients were assigned a number 1 through 3 iteratively in the order of recruitment (Figure 1).

**Figure 2:**
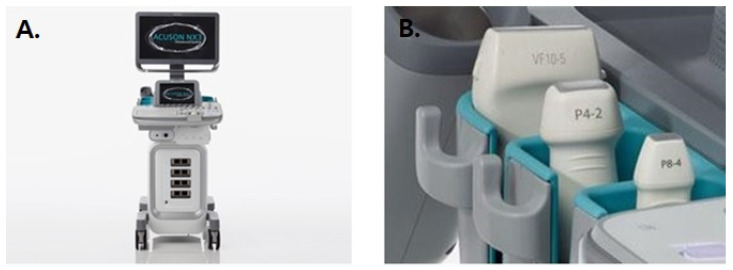
Measurement device (ACUSON NX3 Elite. SIEMENS-Healthineers. Germany). (**A**) Arterial view: ultrasonography device used for the study. (**B**) Probe for measuring Doppler ultrasound.

**Figure 3:**
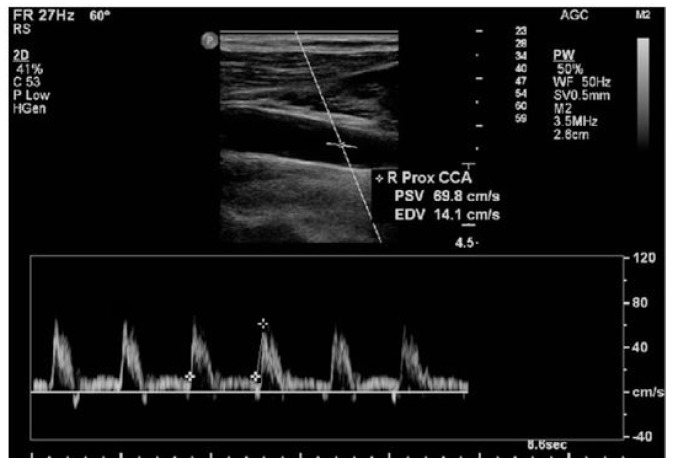
Measurement of ICA by placing a caliper on the level at which the gradient begins to rise at the end of diastole to the first peak of systole (early systolic peak).

EM physicians and EMT-P trained by radiologists performed Doppler ultrasound using ACUSON NX3 (SIEMENS-Healthineers, Germany) (Figure 2). Patients were instructed to lie in a supine position, with the back and head contacting the bed and without bending the neck. The neck was turned slightly in the direction opposite to the carotid artery to test. The ultrasound system was set for a linear transducer and a fundamental frequency ≥ 7 MHz, and the following image setting was chosen: carotid option, depth 4 cm, single focal zone, and frame rate ≥ 25 Hz. The carotid artery on either side was confirmed and the artery diameter was measured. To measure the diameter, the angle between the Doppler beam and the artery was less than 60 degrees and the settings of pulsatile Doppler were chosen. To estimate the inner diameter of the artery in this setup, the size of the diameter was measured by placing the Doppler beam perpendicular to the inner membrane. Afterward, peak systolic and diastolic velocities were measured in area where the intra-carotid arteries were bifurcated, and mean velocity was computed (Figure 3).

To examine vascular reactivity, 2 h after the administration of fluid therapy, ICA diameter, peak systolic and diastolic velocities, and mean velocity were measured again using the same methods as described above [32].

### 2.3. Data Analysis

General characteristics of patients and continuous variables are presented as means ± SDs and categorical variables in %. The primary outcome variable in this study was the accuracy of death risk assessment tools in predicting death. To compare the tools on predictive accuracy, Cox proportional regression analysis was performed, and below, hazard ratios and the 95% confidence intervals are presented. A hazard ratio of 1 was interpreted as no association, a hazard ratio greater than 1 as an increased risk, and a hazard ratio smaller than 1 as a decreased risk. Additionally, the accuracy of death prediction of three measurement tools, qSOFA, SIRS, and ultrasonic Doppler, was compared using Harrell’s C-index. The AUC value was measured through the ROC graph to find out the probability of predicting the death of the ultrasonic Doppler. To determine an optimal cut-off point in predicting death based on mean ICA blood flow velocity, receiver operating characteristic (ROC) curve analysis was performed, and sensitivity and specificity at the cut-off point were computed [33]. The secondary outcome of the this study was to identify the clinical indices influencing the duration of ICU stay in patients. To achieve this objective, univariate logistic regression analysis was performed first, and based on the results, multivariate logistic regression analysis was performed to identify independent factors. Because multivariate regression analysis is based on the assumption that variables are independent of one another, multicollinearity was checked among the variables for which the *p*-value was <0.05 in univariate analysis. Of the variables that were tested for multicollinearity, those with a variance inflation factor value of 10 or higher were determined to be collinear with other variables. Lastly, the tertiary outcome of the study was to demonstrate the usefulness of Doppler ultrasound as a hemodynamic assessment tool by examining changes in mean ICA blood flow velocity and diameter before and after fluid therapy. To do so, a paired-*t*-test was performed to compare ICA blood flow velocities and diameters pre- and post-fluid therapy. Data were analyzed using SAS ver. 9.2 (SAS Institute Inc., Cary, NC, USA) and MedCalc ver. 12.7.0 (MedCalc Software, Ostend, Belgium). If a *p*-value was less than 0.05, the finding was interpreted as statistically significant.

## 3. Results

### 3.1. Characteristics of Subjects

The mean age of the 1026 subjects was 71.3 years ± 9.7; 623 patients (60.7%) were male and 403 patients (39.3%) female. Further, 779 patients (75.9%) had one or more underlying diseases, and 247 patients (24.1%) did not. Regarding the location of the infection, respiratory infection was the most common, affecting 656 patients (63.9%), followed by urinary infection in 235 patients (22.9%). Also, 892 patients (86.9%) had tracheal intubation, and 532 patients (51.8%) died within 30 days; 233 patients (22.8%) survived, and, thus, the number of patients who survived was lower than the number of deaths. Concerning severity classification upon arrival at the ED, on average, the KTAS was 2 ± 0.6 level, the APACHE II score was 32.83 ± 8.23, and the SIRS score was 3.2 ± 1.6. In qSOFA, 913 (88.9%) had a respiratory rate over 22/min, and 1006 (98.1%) had a systolic blood pressure under 100 mmHg. Of these patients, 719 (70.1%) had a GCS under 14 points. Regarding ABGA results, PaO2 was 65.3 ± 32.2 mmHg, PaCO2 was 49.2 ± 21.1 mmHg, and mean base excess in extracellular fluid was −6.3 ± 2.2 mmol/L.

Of the hematologic indices for diagnosing sepsis, the mean CRP was 10 mg/L (5–18), and the mean lactic acid was 2.8 mmol/L (1.1–4.9). Regarding treatments administered following the Surviving Sepsis Campaign guidelines, fluid therapy was administered in 997 (97%) and antibiotics in 1023 (99.7%). A blood culture test was performed in 1024 (99.5%). Further, 102 (10%) subjects were administered antibiotics before arrival at the ED (within 12 h after the onset of illness).

On average, subjects were on mechanical ventilation for 7.36 ± 6.62 days. The mean number of days of ICU stay was 10.57 ± 3.16 days, and the mean number of days of total hospital stay was 17.43 ± 11.33 days (Table 1).

### 3.2. Association Between Clinical Indices and the Duration of ICU Stay

Univariate regression analysis showed that a low level of consciousness, underlying diseases, respiratory infection, tracheal intubation, arterial CO_2_, PETCO_2_, lactic acid level, systolic and diastolic blood pressures, APACHE II score, peak systolic velocity (PSV) in the ICA, and qSOFA score were significant (*p* < 0.05) (Table 2).

A multivariable regression model was constructed, which included the variables showing significant association in univariate regression analysis and multicollinearity was taken into account. The results showed that PSV in the ICA and qSOFA score were associated with the duration of ICU stay, independently of each other (*p* < 0.05) (Table 3).

### 3.3. Association Between Each of the Predictive Tools and Death Within 30 Days

Multivariable logistic regression analysis for predictors was performed by selecting variables that showed significant values through univariable logistic regression analysis. A multivariate Cox proportional hazards model was constructed by including these variables and considering multicollinearity. It was found that PSV in the ICA measured upon arrival at the ED and qSOFA score were associated with death within 30 days (Table 3). In univariate Cox regression analysis on death within 30 days, a low level of consciousness, APACHEII score, lactic acid level, ETCO2, PSV in the ICA, and qSOFA score were found to be associated (*p* < 0.05). The hazard ratio (95% CI) of PSV was 1.020 (1.004–1.036), which was significant (*p* < 0.05). This shows that early measurement of PSV helps to reduce mortality within 30 days. The hazard ratio (95% CI) of qSOFA was 3.871 (2.526–5.931) and it was significant, as well (*p* < 0.05). SIRS showed a hazard ratio (95% CI) of 1.002 (0.995–1.009) and it was significant, as well (*p* < 0.05) (Table 4).

### 3.4. Comparison Between PSV in the ICA and Other Early Risk Assessment Tools on the Predictability of Death

In addition, regarding mortality within 30 days, Harrell’s C-index of PSV in the ICA was 0.862 (95% CI 0.813–0.911), which was significantly higher compared with the other early risk assessment tools (Table 5).

**Figure 4:**
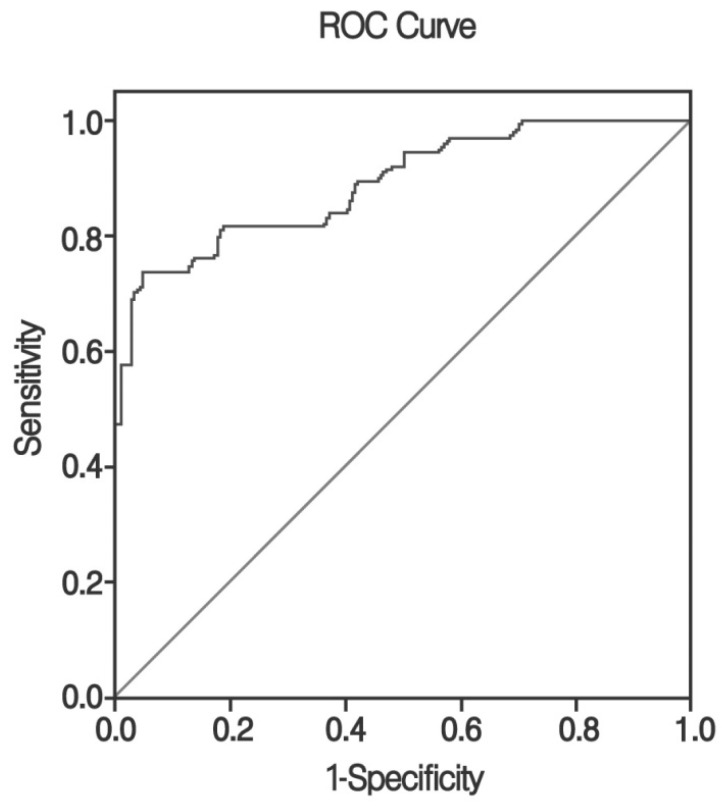
The receiver operating characteristic (ROC) curve of PSV in ICA. AUC (area under the curve) was 0.891 (95% confidence interval 0.826~0.956, *p* < 0.001). PSV: Peak Systolic Velocity; ICA: intra-carotid artery.

An AUC value was computed to examine the predictability of PSV in the ICA. The AUC was 0.891 (95% CI 0.826~0.956), which was statistically significant (Figure 4).

### 3.5. Vascular Reactivity After Fluid Therapy

Changes in vascular reactivity before and after 2 h long fluid therapy are presented in Table 4. The mean ICA diameter was 3.72 ± 0.23 before arrival at the hospital and 4.72 ± 1.23 2 h after fluid therapy, and the difference was significant (*p* < 0.05). Mean PSV was 125 m/s ± 34.21 upon arrival at the ER and 101 m/s ± 21.12 2 h after fluid therapy, and the difference was significant, as well. After fluid therapy, the mean was 4.72 ± 1.23 and the difference was significant (*p* < 0.05) (Table 6).

## 4. Discussion

This study compared SIRS and qSOFA, which are commonly used risk prediction tools for sepsis patients who visited the emergency room, with early ultrasound Doppler ICA diameter size and death prediction accuracy within 30 days of blood flow rate, and we found that early measurement of PSV was useful compared to other measurement tools. And this study showed that the ICA blood flow rate was useful as a mortality predictor through Doppler measurements of carotid ultrasound in sepsis patients, and it was possible to observe changes in the diameter of ICA through initial fluid treatment in sepsis patients due to hypotension. This is because the diameter size of ICA in sepsis patients is narrowed, resulting in increased PSV. This change has the advantage of being able to observe the patient’s physiological changes non-invasively and objectively through the ultrasonic Doppler of sepsis patients. In the late twentieth century, Aaslid et al. introduced transcranial Doppler (TCD) ultrasonography into clinical practice for evaluation of cerebral hemodynamics, ushering in a new era of cerebral circulation on monitoring [33]. Transcranial Doppler (TCD) ultrasonography is a technique that uses a hand-held Doppler transducer (placed on the surface of the cranial skin) to measure the velocity and pulsatility of blood flow within the intracranial and extracranial arteries.

This study showed that mean ICA blood flow velocity measured with Doppler ultrasound in sepsis patients admitted to the ER was highly accurate in predicting death and, thus, its effectiveness as a predictive tool was confirmed. Therefore, it will be possible to propose PSV measurement and ICA diameter size measurement through ultrasonic Doppler for early recognition and response areas currently emphasized in the sepsis algorithm presented by SSC.

In addition, its usefulness as a hemodynamic assessment tool was demonstrated based on the finding that in the presence of hypotension due to hypoperfusion, the ICA diameter expanded after fluid therapy, increasing cerebral blood flow. This study is of significance in that it is the very first study reporting that blood flow velocity, measured with carotid Doppler ultrasound immediately after arrival at the ER, was significantly associated with death in sepsis patients, independently of other variables. Therefore, it will be useful in the clinical field if emergency medical staff who first encounter a patient in the emergency room are trained in early risk recognition methods through ICA measurement rather than early recognition of the patient’s risk through the patient’s vital signs when sepsis patients visit the hospital

In the field of radiology, Doppler has long been proved to be effective as a hemodynamic assessment tool for measuring blood flow. Strosberg DS et al. [34] explained the technical aspects and clinical applications of Doppler. Doppler techniques in medicine to measure blood flow are classified into continuous wave, pulsed wave, and color flow imaging. All of the techniques are based on the fact that the echo frequency of a moving reflector is shifted following the change in frequency of a wave determined by the speed of the source/transducer. Specifically, continuous wave (referred to as CW) Doppler detects the flow at a predefined depth only and is not guided by the image, whereas PW Doppler is performed via dual Doppler technology, in which the velocity and direction of blood flow and the temporal distribution of perturbations in velocity are measured based on the derived curves. In color flow Doppler, Doppler signals in the region of interest are mapped and color-coded to visualize dynamic flow patterns in color. Here, different variables moderating the flow under study are encoded in different colors, making it possible to measure blood flow in each of the blood vessels and perform a hemodynamic assessment. Clinical applications of Doppler based on these mechanisms continue to be researched even today. The effectiveness of Doppler as a hemodynamic assessment tool has been demonstrated in the heart, brain, and major vascular systems, such as carotid arteries, vertebral arteries, and portal veins [33]. Lately, the usefulness of a wireless wearable Doppler as a tool to detect changes in hemodynamics was demonstrated, as well [35].

As highly reliable studies are accumulated in the field of radiology, Doppler is shown to be useful in detecting a change in blood flow due to hypoperfusion.

Recently, several studies were conducted in the field of emergency medicine to investigate the usefulness of ultrasound for assessing patients. However, research has never been performed to examine Doppler as a tool either to predict death in severe patients or to assess hemodynamics. Patients with severe illness or trauma patients commonly visit the ER, and it is crucial to rapidly evaluate, diagnose, and treat them. In particular, compared with patients in other conditions, those with sepsis can survive only if it is recognized early and treated aggressively. So far, the early risk for death of the sepsis patients in the ED has been assessed with the tools created for sepsis patients in the ICU. It is speculated, however, that sepsis patients visiting the ER have different characteristics from those treated in the ICU. Because patients come to the ED via different routes, sepsis may be recognized at a later time in ED patients. Additionally, in sepsis patients in the ED, the source of infection may vary widely, and early recognition is not possible in a situation where there is a large inflow of patients at a given time. Hence, there is a strong need for early recognition of sepsis in the ER, and it is essential to develop a new assessment tool with the characteristics of sepsis patients coming to the ER taken into consideration. In this regard, it is believed that the measurement of blood flow by using carotid Doppler ultrasound would be useful in predicting early risk for death in patients with sepsis or those suspected of sepsis. Studies have shown that the currently used tools, qSOFA and SIRS, are useful as predictive tools regarding the early risk of death in the ED, too. Compared with other tools, the parameters of qSOFA and SIRS can be obtained with only hemodynamic indices, like systolic blood pressure, GCS score, and respiratory rate, but they do have a few limitations. Other variables, such as underlying diseases, intubation rates, and infection sites, are variables that have a significant effect in this study. In particular, since the number of patients with respiratory infections was rapidly increasing in Korea during the study period, sepsis caused by these factors is thought to have had an effect.

In practice, evaluating patient stability by relying on such hemodynamic indices as blood pressure, pulse rate, and respiratory rate is problematic because of compensatory mechanisms in the human body. Accordingly, it seems necessary to develop an assessment tool based on more accurate hemodynamic indices to use in practice.

The comparison of predictability for death among the various tools showed that mean ICA blood flow velocity and diameter were not inferior to qSOFA. Existing high-quality clinical studies have reported that it was quite difficult to use qSOFA or SIRS to assess the severity and predict death in sepsis patients, due to the complicated scoring system and time constraints. In contrast, the Doppler ultrasound of the ICA is more convenient and useful compared to the other scoring systems.

Additionally, this study found that after fluid therapy, ICA blood flow velocity and diameter were significantly increased in comparison to the measurements upon arrival at the ER. Several previous studies have already demonstrated that it was easy to determine the clinical significance of blood flow velocities and artery diameters measured with Doppler. Specifically, Choi et al. [36] examined the association between the extent of carotid artery stenosis and cerebral infarction.

Strosberg et al. [32] stated that, in many situations, the extent of ICA stenosis cannot be determined based on single measurements, pointing out that measurement of ICA can be ambiguous if the angle of the patient’s chin is abnormal, if the ICA is tortuous, or if there is a high level of calcification. Due to these reasons, they argued that predicting the likelihood of ICA stenosis based on the acceleration time of CCA end-diastolic velocity, rather than based on single measurements, may show higher sensitivity. However, it was found in this study that most sepsis patients had 70–99% stenosis, and it was somewhat improved in some patients 2 h after fluid therapy. Though a single measurement of the ICA diameter may have produced an indeterminate finding, it was clear that the size of the diameter increased significantly after fluid therapy. Thus, this study demonstrated that Doppler was a useful hemodynamic assessment tool to monitor the improvement in cerebral blood flow after fluid therapy in sepsis patients. Early fluid therapy for sepsis patients in the ED is recommended by Early Goal-directed Therapy (EGDT). According to the EGDT, fluid and vasopressor therapies are determined based on central venous pressure (CVP), MAP, and central venous oxygen saturation (ScvO2). However, these hemodynamic assessment tools are invasive, and the likelihood of complications is high in severe sepsis patients. Additionally, it is difficult to discern whether blood pressure, heart rate, and respiratory rate returned to normal because blood flow was restored, or the return to normal was merely temporary due to the involvement of compensatory mechanisms. To address these problems, assessing the change in the size of the ICA diameter appears to be a practical, accurate, and easy for monitoring hemodynamics.

This study has a few limitations. First, as it was a single-center study, the findings may not be generalized to the entire sepsis patient population. Second, the proportion of elderly patients was high in the regional emergency medical center where the study was conducted; therefore, the study subjects tended to be older. Finally, we excluded patients who could affect carotid artery size. In other words, the fact that we did not conduct it on all sepsis patients cannot be understood as representing all sepsis patients.

Thus, the study sample may not be representative of all adult patients with sepsis. Third, the blood flow velocity can decrease in the presence of aortic stenosis, but aortic stenosis was not a part of the subject exclusion criteria because it was difficult to diagnose [37].

## 5. Conclusions

It is easy to measure mean ICA blood flow velocity in sepsis patients in the ED to predict the early risk of death. Other sepsis assessment tools rely on the level of consciousness and vital signs, but these cannot be accurately evaluated because of the involvement of compensatory mechanisms. Certain brain hemodynamic patterns emerge during the evolution of sepsis. This trend points to early cerebral vasoconstriction followed by late vasodilatation with increased cerebral blood flow. Accordingly, ICA blood flow velocity is a non-invasive and stable tool to use as an accurate hemodynamic index that can replace the other tools. Furthermore, a change in the size of the intra-carotid artery (ICA) diameter after fluid therapy may serve as a straightforward and accurate method for monitoring the restoration of cerebral blood flow in patients identified with early sepsis.

## Figures and Tables

**Table 1:** Baseline characteristics of study population (N = 1026).

Variables	Values (N (%)) or Mean ± SD
Age (years)	71.3 ± 9.7
Gender	
Male	623 (60.7)
Female	403 (39.3)
Pharmacy history	
Yes	779 (75.9)
No	247 (24.1)
Infection sites (multiple selections, if any)	
Respiratory	656 (63.9)
Genitourinary	235 (22.9)
Gastrointestinal	102 (9.9)
Skin and soft tissue	31 (3)
Others site	2 (0.3)
Insertion Intubation	
Yes	892 (86.9)
No	134 (13.1)
Death (<30 days)	
Yes (death < 30 days)	532 (51.8)
No (death ≥ 30 days)	261 (25.4)
Survive	233 (22.8)
Korean triage and acuity scale (points)	2.2 ± 0.6
Quick SOFA criteria	
RR ≥ 22 (per/min)	913 (88.9)
SBP ≤ 100 (mmHg)	1006 (98.1)
GCS < 14 (points)	719 (70.1)
Pre-ED antibiotics (≤12 h)	102 (10)
ABGA	
PO_2_ (mmHg)	65.3 ± 32.2
PaCO_2_ (mmHg)	49.2 ± 21.1
Mean base excess in extracellular fluid (mmol/L)	−6.3 ± 2.2
PETCO_2_ (mmHg)	31.2 ± 12.2
Biomarkers for sepsis	
CRP (mg/dL)	10 (5–18)
Procalcitonin (ng/mL)	3.2 (0.8–9.7)
Lactate (mmol/L)	3.8 (1.1–5.9)
Body temperature (°C)	38.8 ± 0.6
Systolic blood pressure (mmHg)	90.2 ± 12.1
Diastolic blood pressure (mmHg)	32.1 ± 10.2
qSOFA score (points)	8 (5–11)
APACHE II score (points)	32.83 ± 8.23
Compliance with the SSC bundle	
Fluid resuscitation	997 (97)
Antibiotics	1023 (99.7)
Blood culture	1024 (99.8)
Lactate levels ≥2 times	992 (96.6)
Outcomes	
Duration of MV use (days)	7.36 ± 6.62
ICU length of stay (days)	10.57 ± 3.16
Hospital length of stay (days)	17.43 ± 11.33

Values are presented as median (interquartile range, IQR), number (%), or mean ± SD. qSOFA: quick Sequential Organ Failure Assessment; RR: respiratory rate; SBP, systolic blood pressure; GCS: Glasgow Coma Scale; ETCO: end Tidal carbon dioxide; MV: mechanical ventilator; APACHE II score: the acute physiology, and chronic health evaluation II score; ED: emergency department; CRP: C-reactive protein; SSC: surviving sepsis campaign; IQR: interquartile range; SD: standard deviations; ICU: intensive care unit.

**Table 2:** Univariable logistic regression analysis for predictors of the period of ICU stay (n = 1026).

Variable	Period of ICU Stay	
	OR (95% CI)	*p*-Value
Age	1.006 (0.991–1.021)	0.432
Gender	1.508 (0.773–2.942)	0.228
Pharmacy history	0.979 (0.965–0.994)	<0.001
Infection of respiratory	2.044 (1.156–3.617)	<0.001
Insertion Intubation	1.980 (1.037–3.779)	0.038
Korean triage acuity scale (points)	1.008 (0.996–1.021)	0.279
GCS < 14 (points)	5.693 (3.009–10.770)	<0.001
Pre-ED antibiotics (≤12 h)	0.990 (0.947–1.036)	0.673
ABGA		
PO_2_ (mmHg)	1.326 (0.956–1.838)	0.091
PaCO_2_ (mmHg)	0.451 (0.295–0.689)	<0.001
Mean base excess in extracellular fluid (mmol/L)	0.903 (0.544–1.497)	0.692
ETCO_2_ (mmHg)	0.850 (0.784–0.921)	<0.001
Biomarkers for sepsis		
CRP (mg/dL)	0.990 (0.947–1.036)	0.673
Procalcitonin (ng/mL)	0.917 (0.105–7.999)	0.938
Lactate (mmol/L)	1.308 (1.187–1.442)	<0.001
Body temperature (°C)	0.297 (0.014–6.404)	0.438
Systolic blood pressure (mmHg)	0.984 (0.975–0.993)	<0.001
Diastolic blood pressure (mmHg)	0.979 (0.965–0.994)	0.006
qSOFA score (point)	1.736 (1.526–1.974)	<0.001
APACHE II score (point)	1.150 (1.101–1.202)	<0.001
PSV in ICA (cm/s)	71.2 (65.232–77.232)	<0.001
Vmean (cm/s)	67.2 (61.102–75.511)	0.279
Diameter of ICA (cm)	5.580 (4.551–6.133)	0.395

ICU: intensive care unit; OR: Odds Ratio; CI: Confidence Interval; GCS: Glasgow Coma Scale; ED: emergency department; ETCO_2_: End Tidal Carbon dioxide; CRP: C-reactive protein; qSOFA: quick sequential organ failure assessment; APACHE II score: the acute physiology, and chronic health evaluation II score; ICA: intra-carotid Artery; PSV: Peak Systolic Velocity; Vmean: mean velocity.

**Table 3:** Multivariable logistic regression analysis for predictors of the period of ICU stay.

Variables	Period of ICU Stay	
	OR(95% CI)	*p*-Value
Pharmacy history	1.006 (0.991–1.021)	0.432
Infection of respiratory	1.508 (0.773–2.942)	0.228
Insertion Intubation	1.074 (0.209–5.524)	0.932
GCS < 14 (points)	2.071 (0.915–4.686)	0.081
PACO_2_ (mmHg)	1.008 (0.996–1.021)	0.279
ETCO_2_ (mmHg)	1.014 (0.903–1.140)	0.81
qSOFA score (points)	1.019 (1.000–1.038)	<0.048
Systolic blood pressure (mmHg)	1.171 (0.781–1.755)	0.445
Diastolic blood pressure (mmHg)	0.567 (0.061–5.277)	0.618
APACHE II score	1.499 (0.641–3.506)	0.35
PSV in ICA (cm/s)	1.078 (1.042–1.115)	<0.001

ICU: intensive care unit; OR: odds ratio; CI: confidence Interval; GCS: Glasgow Coma Scale; ETCO_2_: end tidal carbon dioxide; APACHE II score: the acute physiology and chronic health evaluation II score; ICA: intra-carotid Artery; PSV: peak systolic velocity.

**Table 4:** Univariable Cox proportional hazard regression analysis for predictors of 30-day mortality.

Variables	Hazard Ratio (95% CI)	*p*-Value
PSV in ICA (cm/s)	1.020 (1.004–1.036)	<0.001
qSOFA (points)	3.871 (2.526–5.931)	<0.001
SIRS (points)	1.002 (0.995–1.009)	0.021
APACHE II score (points)	1.150 (1.113–1.187)	0.216
GCS < 14 (points)	05.633 (2.948–10.762)	0.491
Lactate (mmol/L)	1.233 (1.159–1.313)	0.08
ETCO_2_ (mmHg)	0.854 (0.791–0.921)	0.187

CI: confidence interval; ICA: Intra-carotid Artery; PSV: peak systolic velocity; qSOFA: quick sequential organ failure; SIRS criteria: systemic inflammatory response syndrome criteria; APACHE II score: the acute physiology and chronic health evaluation II score; GCS: Glasgow Coma Scale; ETCO_2_: end tidal carbon dioxide. HR: the ratio of the hazard rates corresponding to the conditions characterised by two distinct levels of a treatment variable of interest.

**Table 5:** Comparing scoring systems and PSV in ICA for the prediction of 30-day mortality using Harrell‘s C-index.

Variables	Harrell’s C-Index (95% CI)	*p*-Value	*p*-Value (vs. PSV)	*p*-Value (vs. qSOFA)	*p*-Value (vs. SIRS)
SIRS	0.691 (0.589–0.793)	<0.001	Reference	0.791	0.079
qSOFA	0.796 (0.729–0.863)	<0.001	0.791	Reference	0.091
PSV in ICA	0.862 (0.813–0.911)	0.002	0.079	0.091	Reference

CI: confidence interval; ICA, Intra-carotid Artery; PSV, Peak Systolic Velocity; qSOFA, Quick Sequential Organ Failure; SIRS criteria, systemic inflammatory response syndrome criteria.

**Table 6:** Compare with a change in diameter of ICA after 2 h carryout fluid therapy.

Variable	Initial Phase	After 2 h Phase (After Fluid Therapy)	*p*-Value
PSV in ICA (cm/s)	125 ± 34.21	101 m/s ± 21.12	<0.001
Diameter of ICA (cm)	3.72 ± 0.23	4.72 ± 1.23	<0.001

ICA: intra-carotid Artery; PSV: peak systolic velocity.

## Data Availability

The data that support the findings of this study are available from the corresponding author (Y.D.J.) upon reasonable request.
